# Relevance of Drift Components and Unit-to-Unit Variability in the Predictive Maintenance of Low-Cost Electrochemical Sensor Systems in Air Quality Monitoring

**DOI:** 10.3390/s21093298

**Published:** 2021-05-10

**Authors:** Georgi Tancev

**Affiliations:** Swiss Federal Institute of Metrology, 3084 Bern, Switzerland; georgi.tancev@metas.ch

**Keywords:** air quality monitoring, anomaly detection, gas sensor, low-cost sensors, machine learning, predictive maintenance

## Abstract

As key components of low-cost sensor systems in air quality monitoring, electrochemical gas sensors have recently received a lot of interest but suffer from unit-to-unit variability and different drift components such as aging and concept drift, depending on the calibration approach. Magnitudes of drift can vary across sensors of the same type, and uniform recalibration intervals might lead to insufficient performance for some sensors. This publication evaluates the opportunity to perform predictive maintenance solely by the use of calibration data, thereby detecting the optimal moment for recalibration and improving recalibration intervals and measurement results. Specifically, the idea is to define confidence regions around the calibration data and to monitor the relative position of incoming sensor signals during operation. The emphasis lies on four algorithms from unsupervised anomaly detection—namely, robust covariance, local outlier factor, one-class support vector machine, and isolation forest. Moreover, the behavior of unit-to-unit variability and various drift components on the performance of the algorithms is discussed by analyzing published field experiments and by performing Monte Carlo simulations based on sensing and aging models. Although unsupervised anomaly detection on calibration data can disclose the reliability of measurement results, simulation results suggest that this does not translate to every sensor system due to unfavorable arrangements of baseline drifts paired with sensitivity drift.

## 1. Introduction

Due to the known adverse effects of air pollution, cities consider monitoring relevant air quality indices a necessity [1,2,3]. Studies report that many premature deaths could be prevented by complying with air quality guidelines such as the ones provided by the World Health Organization [4]. To satisfy the desire for higher spatial and temporal resolution due to differences in individual exposure [5], a lot of research on affordable air quality monitoring devices has been performed [6,7,8,9,10,11,12,13,14,15]. Such devices consist of gas and/or particulate matter sensors in the low-cost range, and they are supposed to be connected to the internet of things, thus forming a network and being part of the smart city vision [16]. More often than not, these low-cost systems contain data-derived models obtained from machine learning (ML) algorithms, a subfield of artificial intelligence, to compensate for cross-sensitivities and/or interferences with environmental factors (temperature, humidity, pressure) of the sensors, with the aim to decrease the uncertainty of measurement results and increase the value of the product.

While low-cost particulate matter sensors immediately provide interpretable output, this is generally not the case for gas sensors, as they only deliver raw sensor signal intensity—e.g., voltage or current, thereby requiring calibration [17,18,19]. As mentioned above, change in raw signal *s* is not only caused by the target gas *r*_1_, but also by other gases or environmental factors *r*_2_, … *r_n_*, which motivated use of a multivariate approach via sensor fusion [11]. The amount by which each variable affects the sensor signal is described by its parameters *α_k_* belonging to the individual factors. Therefore, a gas sensor generates signals according to a sensing model *f* (Equation (1)). Note that Equation (1) is usually inverted to compute the fractions of the target compounds.

(1) $${s=f(r}_{1}{,\;r}_{2}{,\;\ldots ,\;r}_{n}{;\;\alpha }_{0}{,\;\alpha }_{1}{,\;\ldots ,\;\alpha }_{n})$$

In reality, every low-cost sensor has a different baseline (zero), responds differently to the gases and environmental factors, and adds noise to the signal, thereby requiring individual calibration [20,21,22,23]. Due to this high unit-to-unit variability (in combination with drift due to aging, i.e., time-dependent parameters *α_k_*), laboratory calibration is currently considered too expensive [11]. Hence, most low-cost sensor systems are characterized with field data obtained by collocating devices next to reference stations [6,7,13]. This data can contain many nonlinear relationships, partly due to the sensor responses, but also due to the complex relationships between the interfering variables [13]. 

Newer studies have shown that these models do not generalize well due to strong correlations within the field data, and the devices suffer from spatial and temporal relocation problems [24,25]. The underlying cause is the difference in probability distributions of the factors across different locations and times, known as concept drift. It is debatable if such systems could ever be certified under these circumstances, since they “replay” seen relationships and perform only quasi-measurements. In particular, the measurement uncertainty can change over time and space, thus results can become unreliable under changing atmospheric conditions. Ideally, the data acquisition process should be designed in a way to yield independent (orthogonal) variables using some design of experiments [26]. This is particularly relevant if the data quality objective of legislators should be met, as air quality data could not be used for decision-making otherwise. Nonetheless, it boils down to the role of low-cost sensor systems and what purpose they should fulfill in the future [27].

A recent publication suggested to proceed with field calibration and to compare multivariate distributions of pollutants and environmental conditions (i.e., the atmosphere) across locations in order to decide the suitability of a field-calibrated device for a specific location [25]. In this case, the comparison of distributions is achieved by using similarity metrics for probability distributions, one of them being the Kullback–Leibler divergence *D_KL_* (Equation (2)), which is a measure of how one probability distribution *p*(*x*) is different from a reference probability distribution *q*(*x*) [28,29].

(2) $$D_{KL}(p||q)=\int_{-\infty }^{\infty }p(x)log\frac{p(x)}{q(x)}dx$$

More precisely, *q*(*x*) is the distribution from the calibration data, and *p*(*x*) is the operation distribution at a new location. Furthermore, the authors mention that such metrics could serve for dynamic recalibration procedures, so that low-cost air quality monitors will require service if metrics exceed some threshold. This methodology is known as predictive maintenance (PD) [30]. Nonetheless, it is worth noting that different drift components, e.g., aging and concept drift, will overlap with field calibration, and it cannot be known whether a sensor has aged, or the temporally or spatially local atmosphere has changed (e.g., all points that are measured during a certain period at a certain place). Thus, recalibration intervals can be shorter with field calibration due to this additional drift component.

Unfortunately, the proposed approach lacks practicability, as in most cases the atmospheric composition of a specific location will not be known in advance in order to decide whether a performed calibration is suitable or not. Therefore, it would be more reasonable to focus on a method that relies only on the calibration data. Additionally, choosing a threshold of the Kullback–Leibler divergence is not an intuitive task, since it is bound between zero and infinity and hard to interpret. Theoretically, it is also necessary to refit the distribution *p*(*x*) with every newly acquired data point, followed by numerical computation of the integral (unless the data come from a generic distribution, e.g., standard normal, for which closed-form solutions exist).

Nonetheless, PD has been applied on several occasions and bears enormous potential [30]. For low-cost sensors, the interest usually revolved around the removal/autocorrection of drift components instead [31,32,33]. In the context of metrology, it enables dynamic service of devices as opposed to fixed predefined maintenance intervals. This is particularly relevant, as the unit-to-unit variability of low-cost sensors is significant and a fixed recalibration interval, e.g., with transfer standards, for all devices cannot be considered a satisfying solution [34]. Therefore, the reliability of measurement results can be increased. Furthermore, remote devices can call for service autonomously as soon as some of their parameters show a trend towards malfunction. In addition, it can balance workload, as devices with similar needs for maintenance can be grouped together for parallel recalibration. For networks of such instruments, any optimization of operation cost leads to cost reduction.

Detecting unusual behavior, e.g., system malfunction, is a common ML task in which normal and non-normal operation states have to be distinguished—a problem known as anomaly detection [35,36]. In contrast to the proposed approach using the Kullback–Leibler divergence, there is a subset of techniques that requires only data from the calibration procedure (which is available anyway), but not from the location where the device would be placed at [37,38,39,40]. Low-cost sensors are consumables and degrade over time due to aging. Therefore, one could hypothesize that sensors move away from their reference states that are defined by the fresh sample. The reference state can be thought of as a multivariate probability distribution (“generated” by the set of sensor signals of a brand-new system that is exposed to different conditions); over time, this distribution changes its parameters, i.e., mean and/or covariance, due to aging.

**Figure 1:**
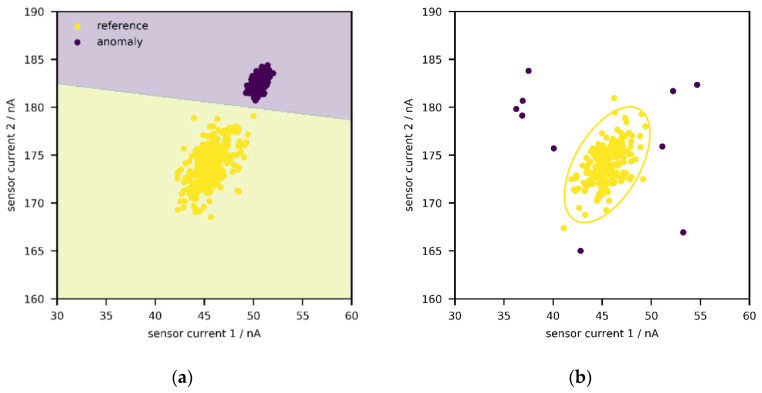
Distribution of sensor currents for a system consisting of two sensors. (**a**) Classification of anomalies in signal space with supervised anomaly detection. (**b**) Classification of anomalies in signal space with unsupervised anomaly detection.

In the following, two general approaches shall briefly be sketched. First, with supervised anomaly detection (Figure 1a), one or more classes of anomalies can be directly targeted as long as labelled data are available [30,35,36]. In some cases, such data can be obtained by performing stress tests—e.g., by exposing a machine/device to extreme environments or operating under unusual conditions for some time in laboratory environments. In Figure 1a, the reference state and one anomaly class are depicted; a support-vector machine with linear kernel is used to describe the decision boundary between the two states, but any other classification algorithm works just as well—e.g., logistic regression, random forest, neural network.

Unfortunately, it is rarely known in advance what non-normal (or aged) states look like; even if historical data from stress testing might be available, it is likely incomplete so that not every possible defect has been captured. Therefore, it could be more interesting to just identify every point outside a reference state, independent of its direction (Figure 1b) [30,35,36]. This is known as unsupervised anomaly detection, and this publication will explore the suitability of such an approach. One archetype of methods among the plethora of algorithms aims to construct an envelope around the data, i.e., the set of points from the reference state, so that points outside of it can be identified (Figure 1b). Such an envelope can be interpreted as a confidence region, and newly generated points are regarded as “acceptable” if they lie within said region. As implied above, the reference state is generated during the calibration procedure according to Equation (1), by exposing a system to different concentrations of gases and environmental conditions. Due to aging or concept drift, one could expect that, over time, more and more sensor signals would fall outside the envelope. In this manner, the need for recalibration (or the suitability of a calibration) could be identified during operation.

As an example, one could fit a normal distribution to the data set, and in a second step, compute the Mahalanobis distance (the extension of the *Z*-score to more than one dimension) of points to estimate their regularity [37]. However, the actual distribution of the data does not need to be normal, and in that case, other techniques can be applied with the same idea—e.g., local-outlier factor [38], one-class support vector machine [39], or isolation forest [40]. The common concept is to identify regions in the feature/signal space where the probability distribution “lives” or resides. Note that the regions do not need to be contiguous—e.g., in the presence of multiple clusters. Such methods do not necessarily compute a distance metric but rely on either an anomaly score or the position relative to the envelope (inside/outside); moreover, they allow defining an initial amount of outliers/contamination *ν* to control the shape/tightness of the volume, which is a hyperparameter. Furthermore, if there are already some outliers in the training set, the algorithms will try to identify them by itself.

Nonetheless, there are several aspects to keep in mind in such applications. Differences in available algorithms have to be investigated on a standardized data set in order to compare them. Moreover, the physics of aging drift and unit-to-unit variability have to be studied, since they might affect the viability of the methodology. Besides, if calibration occurs on the field, additional drift components can interfere with aging drift, and the recalibration intervals can end up being rather short. This work aimed to address these points by expanding the PD approach with an emphasis on electrochemical gas sensors and unsupervised anomaly detection. To study the suitability of the methodology, a sensing and aging drift model was developed. The benefit of having such a model is that Monte Carlo simulations of laboratory calibration followed by aging can be performed and compared with published experimental (field) data, thus accounting for the unit-to-unit variability of sensors and different drift components.

## 2. Materials and Methods

In the following section, one robust parametric method from statistics, the Mahalanobis distance, and three nonparametric ML algorithms are briefly outlined (nonparametric methods do not assume any kind of distribution and construct envelopes of any shape). The reader is encouraged to consult fundamental ML literature, as the presented algorithms are essentially extensions or modifications of existing algorithms such as *k*-nearest neighbors, support vector machine, or random forest [28,29]. 

Common to the ML techniques is the presence of hyperparameters—i.e., variables that control the behavior of fitted models. In supervised scenarios, these parameters are optimized with objective functions, e.g., mean squared error/accuracy, which is not possible in unsupervised learning as the ground truth is not given. Where available, recommendations from the academic literature have been considered. However, the most important hyperparameter is the initial amount of contamination *ν* in the training data, whose influence has to be evaluated. All applied algorithms are implemented in *Scikit-Learn*, an open-source library for ML in Python [41].

### 2.1. Robust Covariance (Mahalanobis Distance)

The generalization of the *Z*-score for a point *x_i_* in the case of a multivariate normal distribution with some mean *µ* and covariance matrix *Σ* is known as Mahalanobis distance, which is given by Equation (3).

(3) $$d_{i}=\sqrt{{{(x}_{i}-\mu )}^{T}\Sigma^{-1}{(x}_{i}-\mu )}$$

It is based on the idea of measuring how many standard deviations *σ* away *x_i_* is from *µ*. An extreme observation has a large distance from the center of a normal distribution. Given a data set ***X*** with *n* samples in *p* variables, mean and covariance matrix are easily computed. However, in the presence of outliers/anomalies, both estimates are distorted and the Mahalanobis distance is rendered useless; hence, a so-called “robust” method would be desired. Robust covariance (RC) methods are based on the idea that outliers lead to an increase in the values (entries) in the covariance matrix, making the spread of the data apparently larger. Consequently, the determinant |*Σ*| will also be larger, which would theoretically decrease by removing extreme events. Rousseeuw and Van Driessen developed a computationally efficient algorithm that yields robust covariance estimates [37]. The method is based on the assumption that at least *h* out of the *n* samples are regular (*h* is a hyperparameter), whereas the relationship between the fraction of contamination and *h* is given by *ν* = 1 − *hn^−^*^1^.

The algorithm starts with *k* random samples, each with (*p* + 1) points. For every sample, μ, *Σ*, and |*Σ*| are estimated, all distances are calculated, sorted in increasing order, and the *h* smallest distances are used to recompute the estimates. In their original publication, the subroutine of computing distances and updating the estimates is called a “C-step”. They figured out that two such steps are sufficient to find good candidates for *µ* and *Σ* among the *k* random samples. In a next step, a subset *m* out of *k* with the lowest |*Σ*| is considered for computation until convergence (the *k* best candidates), and the estimates belonging to the lowest |*Σ*| are returned as output.

In general, the squared Mahalanobis distance *d^2^* follows a *Χ_p_*^2^ (“chi-square”) distribution with *p* degrees of freedom. To compute a critical distance (threshold) for the envelope, a quantile, i.e., the probability of observing a squared distance as extreme as the threshold, the *p*-value, is chosen and the critical distance is then computed from the inverse cumulative distribution. As an example, for one single variable and a probability threshold/*p*-value of 0.05, the critical *Χ*_1_^2^-square value is 3.84, which corresponds to *d*^2^; the square root of that is 1.96, which is consistent with the intuition for one dimension—e.g., from statistical tests. (Note that this is solved differently in *Scikit-Learn*; the threshold is the largest distance retained—i.e., the largest distance that is still part of the robust covariance estimate; the effect on the outcome is negligible.)

### 2.2. Local Outlier Factor

The local outlier factor (LOF) is a modification of the *k*-nearest neighbor algorithm that requires no labelled data [38]. The *k*-nearest neighbor algorithm is a supervised learning technique that bases predictions (for test points) on the closest *k* points (e.g., with respect to Euclidean distance) in the training data, whereas *k* is a hyperparameter. The training phase is very simple, depositing all data, labels inclusive, in a database or random-access memory. For a prediction on a new point, the distances to all training points are computed and sorted in increasing order. Then, the labels of the smallest *k* distances are counted (in classification tasks) and the label with the highest count is returned as a prediction. 

On average, anomalies have larger distances to all other points (low density) compared to regular points. By comparing the local density of a point to the local densities of its neighbors, the algorithm can identify regions of similar or higher densities and points that have significantly lower densities than their neighbors, thereby considering them as anomalies/outliers. The LOF is the output of the algorithm and, as such, is an anomaly score, between zero and infinity, whereas scores around and above one are outliers rather than inliers (the opposite being true for scores below one), but no well-defined thresholds exist. Therefore, the number of contaminations can be used to determine this threshold by evaluating the scores within the training data, similar to the robust covariance approach.

Methods for choosing optimal values for *k* have been elaborated in the original publication; the authors proposed *k*-values above 10 but lower than the number of points as part of distinct clusters with the goal of retaining the structure of these clusters. In the application here, the idea is to have only one single cluster; hence, *k* was set to 200 (two days of quarter-hourly or eight days of hourly calibration data).

### 2.3. One-Class Support Vector Machine

The one-class support vector machine (OCSVM) algorithm is an extension of the regular support vector machine, which itself is a method from supervised learning [39]. A support vector machine tries to separate one class from all other classes by the means of a hyperplane, and it is assessed on which side of the hyperplane points lie. However, there might be many potential hyperplanes, e.g., by shifting or rotating, so the one with the largest distance (maximum margin) to the closest pair of points on both sides is chosen. In reality, classes will never be completely separable, particularly not with a linear decision boundary. Hence, two refinements had to be made; on one hand, points of each class are allowed to cross decision boundaries, whereas the amount of this “line-crossing” is controlled by a hyperparameter (directly related to the amount of contamination in the one-class version). On the other hand, instead of working in the original feature space, the data are “mapped” to another space of higher dimension via basis expansion, which is implicitly performed with a so-called kernel. Different types of kernels exist that contain hyperparameters themselves as well; in the following, a radial basis kernel with hyperparameter *γ* = *p^−^*^1^ is applied, *p* being the number of variables within the respective data set (in *Scikit-Learn*, this is accomplished by setting the kernel parameter to “auto”).

The extension of the support vector machine to an unsupervised method is based on the idea of separating all points from the origin, which leads to the construction of an envelope around the data, whereas it is possible to account for contamination in the training data.

### 2.4. Isolation Forest

The isolation forest (IF) is a modification of the random forest for unlabeled data in anomaly detection [40]. Both algorithms construct decision trees during the training phase, but while decision trees in a random forest try to separate (or isolate) points of the same class by optimizing some function, e.g., Gini index, the IF aims to separate all points from each other by random splits. On average, extreme points are more easily separated and are found closer to the root of a decision tree, thereby having shorter paths from the root, while the opposite is true for regular points. Finally, the expected path length of a point across all trees is used to compute an anomaly score that is bound between zero and one, which is closer to unity if an instance is considered extreme.

During the test time, a point is passed through all trees and the anomaly score is computed; if it is above some predefined threshold, for instance, one determined by the amount of contamination in the training data, it can be considered an anomaly. Note that the number of trees in a forest is a hyperparameter, and the original publication reports that values above 100 do not necessarily exhibit better performances, so this value was applied here as well [40].

### 2.5. Data

Data management and wrangling was performed using *Pandas*, an open-source library for data manipulation in Python [42]. Field data from Zimmerman et al., were used for the analysis, which were collected at an urban background site [10]. The data consist, among other things, of measurements from several devices with built-in Alphasense gas sensors for carbon monoxide (CO), carbon dioxide (CO_2_), nitrogen dioxide (NO_2_), ozone (O_3_/NO_2_ combined), and sulfur dioxide (SO_2_), as well as temperature and relative humidity sensors, in four data points per hour over a period of six months (August to February). The fraction references of CO, CO_2_, NO_2_, and O_3_ in nmol/mol, also known as parts-per-billion (ppb), are available starting from the third month. Thus, only this period was analyzed. The three devices with the lowest amount of missing data were retained (device 1: #4, device 2: #16, device 3: #17). The global structure of the data is shown in Figure S1 (starting from the third month). 

The analysis was centered on the three pollutants CO, NO_2_, and O_3_ (CO_2_ and SO_2_ have been omitted). The properties of the corresponding sensors are listed in Table 1 [20,21,22]. Before analysis, the data set was preprocessed by imputing missing values with averages for each time trace, followed by scaling values to the range between zero and one. As in a hypothetical real-world application, training was performed in the first two weeks of the data using the aforementioned time traces, whereas monitoring/validation was carried out on the residual data (separately for each device and the reference data).

### 2.6. Sensing and Aging Drift Model

In order to analyze the effects of unit-to-unit variability and different drift components on the performance of the applied algorithms, a deterministic sensing and aging drift model was implemented (based on the Python library *NumPy* [43]). The sensing model from Equation (1) was reduced to one target gas (no interferences) with one sensitivity coefficient and a baseline (zero). The exception is the OX-B4 sensor, which is a combined sensor for O_3_ and NO_2_ with shared coefficients. Hence, only the target gases are affecting the signals of the *i*-th sensor in this model. The model structure and its coefficients are in line with the data from the manufacturer (Table 1). In Equation (4), *s_i_* is generated by target gas *r_i_* with sensitivity *α*_1,*i*_ and baseline/zero *α*_0,*i*_.

(4) $$s_{i}{=\alpha }_{1,i}r_{i}{+\alpha }_{0,i}$$

Furthermore, both coefficients were taken as time-dependent. The drift δ for the sensitivity is an exponential decay (Equation (5)) and for the baseline a linear shift (Equation (6)). Since the drift is given over a year, it is adjusted for different step sizes *n*. (For instance, if the discrete time steps are days, *n* equals 365.)

(5) $$\alpha_{1,i}^{t}{=\alpha }_{1,i}^{0}\partial_{1,i}^{\frac{t}{n}}$$

(6) $$\alpha_{0,i}^{t}{=\alpha }_{0,i}^{0}+\partial_{0,i}\frac{t}{n}$$

To account for sensor variability, the coefficients *α*_*k*,*i*_^0^ and *δ*_*k*,*i*_ were sampled from uniform distributions *U* (Equations (7) and (8)), parametrized by the lower and upper bounds of the entries in Table 1.

(7) $$\alpha_{k,i}^{0}\;∼{\;U(lb}_{\alpha_{k,i}^{0}}{,\;ub}_{\alpha_{k,i}^{0}})$$

(8) $$\partial_{k,i}\;∼{\;U(lb}_{\partial_{k,i}}{,\;ub}_{\partial_{k,i}})$$

In a typical Monte Carlo experiment, a virtual device with all three sensors was created by sampling all parameters. To simulate a laboratory calibration at *t* = 0, the device (with its model parameters) was “exposed” to the target gases whose fractions are sampled from uniform distributions (number of values as well as upper and lower bounds specified where necessary), which then generated a distribution over sensors signals for one specific device. This guarantees that all variables are independent and uncorrelated. Gaussian noise with zero mean and 2 nA standard deviation was superimposed to the signals [20,21,22]. By increasing the time index, the sensitivity and zero coefficients were tracked and the behavior of the sensors monitored.

## 3. Results and Discussion

**Figure 2:**
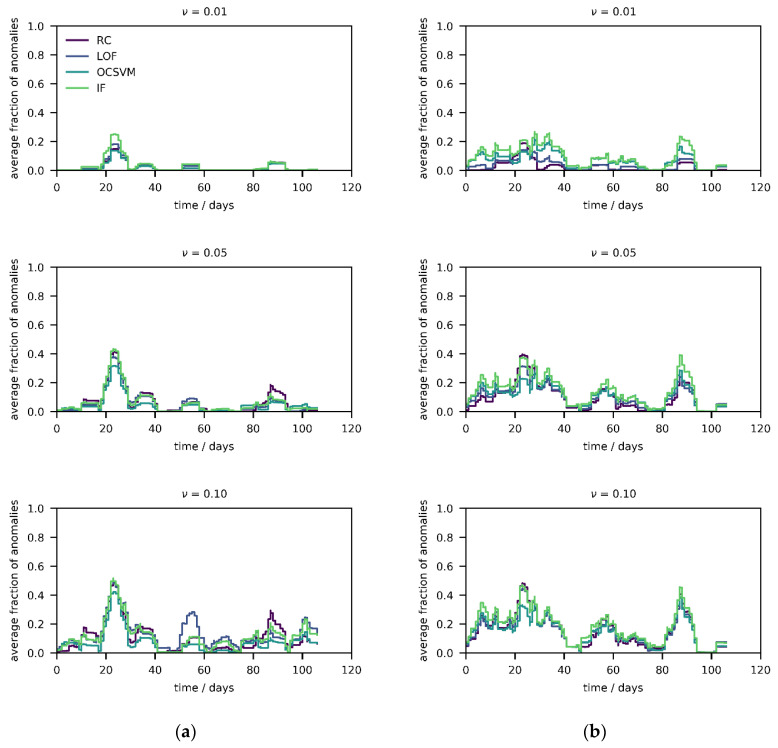
(**a**) Fraction of anomalies (weekly moving average) over four months for different *ν* across algorithms after two weeks of field calibration for sensor data of device 1. (**b**) Fraction of anomalies (weekly moving average) over four months for different *ν* across algorithms after two weeks of field calibration for reference data.

In a first step, the four algorithms were tested on the published field experiments, thereby modelling field calibration, with three values of *ν* (0.01, 0.05, and 0.10). In Figure 2, results from device 1 (which has the fewest missing values) and reference data are presented. More precisely, the average fraction of anomalies (weekly moving average, centered on every time point) has been computed and a few interesting observations are made. First, the output of the four algorithms is consistent and the curves are overlapping, thereby suggesting (close to) normally distributed data, as otherwise the RC model would deviate from the nonparametric methods; this is true for both sensor (Figure 2a) and reference data (Figure 2b). Moreover, the curves of the reference data match with the one of the sensor data, which suggests that the generated anomalies are a result from concept drift and a change in the underlying distribution, i.e., conditions that have not been observed during the calibration phase, as the references would not contain any anomalies otherwise. Such “anomalous” conditions include higher or lower values of either pollutant(s) but also different combinations compared to the ones in during the calibration phase. Thus, the measurement results can be completely unreliable during these periods—for instance, if the calibration function is extrapolated (e.g., random forests cannot extrapolate at all) [24]. Something that is not necessarily captured is the rotation of the probability distribution (i.e., changing entries in the covariance matrix) of the current atmosphere due to varying relationships between the pollutants [24]. Unfortunately, devices 2 and 3 have more missing information compared to device 1, but the aforementioned trends appear to be the same (Figure S2). The average fraction of anomalies increases with increasing values of *ν*, mostly between 0.01 and 0.05, but halts after that. The smaller the value of *ν*, the larger the envelope, as less initial anomalies/outliers are removed during the construction of the envelopes. Hence, for very low values, almost no anomalies will be detected, and hyperparameter *ν* should be increased unless the profile stops changing (quasi-convergence).

Interestingly, there are only few anomalies at the end of the four months of operation, both in the sensor and reference data, suggesting that the aging process might not yet be significant over this period. It would have been more intuitive if the frequency of anomalies would increase over time, and it is remarkable that the average fraction of anomalies only increases and decreases periodically, which can be attributed to the field calibration as explained above. Therefore, such anomaly scores disclose the reliability of the measurement results generated by field calibrated air quality monitors, which could be considered as safety measure in future applications.

To understand which pollutants are responsible for the anomalies, the quantiles of the calibration reference data inside the envelope (c) were compared against the quantiles of the monitoring data outside the envelope (m), summarized in Table 2. The analysis shows that, during operation, the gas levels are either higher (CO, NO_2_) or lower (O_3_), which then causes the increase in anomalies in Figure 2. This concept drift is a consequence of field calibration [24], as more combustion for heating in the winter leads to higher levels of CO and NO_2_, while less radiation leads to a decrease in O_3_ levels. Nonetheless, these spikes in the anomaly score can be prevented by extending the calibration range in a laboratory setting.

The measurement period can be considered comparably short, so a longer study might be needed. For this reason, the data set from De Vito et al. [6], which has been characterized in an earlier publication [24], was also tested under the same procedure (two weeks of field calibration with sensors for CO, NO_2_, and O_3_ from a different manufacturer); the results are depicted in Figure S3. Note that the values for *ν* had to be increased to reach “convergence”. With these data, the parametric method diverges from the nonparametric methods. While the nonparametric techniques agree in most scenarios, they disagree in the sensor data at moderate to high values of *ν*. Figure S3a,b demonstrate the increase in the fraction of anomalies over time, both in the sensor and reference data. This illustrates a late starting concept drift, as the atmosphere changes continuously over time. Nonetheless, the increase in the sensor data appears steeper, hinting at an additional drift component (e.g., resulting from aging).

**Figure 3:**
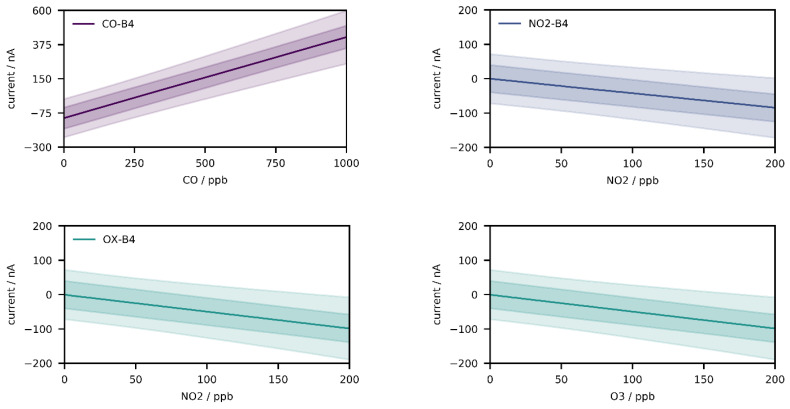
Distribution of the three sensor populations (q_0.50_, q_0.25–0.75_, q_0.05–0.95_) with respect to zero (baseline) and sensitivity by exposure to 200 evenly spaced gas fractions (CO: 0–1000 ppb, NO_2_: 0–200 ppb, O_3_: 0–200 ppb) without mixing.

Thus far, these results seem promising, but they are obtained only from a few field-calibrated devices. To examine the performance on the population without concept drift, Monte Carlo experiments were performed. In a first step, the implemented population model was inspected to prove its correctness. For this purpose, 10,000 randomly sampled devices were exposed to 200 evenly spaced gas fractions (CO: 0–1000 ppb, NO_2_: 0–200 ppb, O_3_: 0–200 ppb) without mixing, and Figure 3 depicts the results of the calibration simulation (q_0.50,_ q_0.25–0.75_, q_0.05–0.95_) of the three sensors as function of the fractions. The computed median baselines (CO-B4: −110.45 nA, NO_2_-B4: 0.41 nA, OX-B4: −0.79 nA) match approximately the median baselines in Table 1, which is also true for the median sensitivities (CO-B4: 0.53 nA/ppb, NO_2_-B4: −0.43 nA/ppb, OX-B4: −0.49 nA/ppb), and deviations result from the Monte Carlo error.

In Figure S4, a randomly sampled device was calibrated (with 10,000 randomly sampled values) in two different settings, once with lower fractions of NO_2_ (Figure S4a, CO: 0–400 ppb, NO_2_: 0–15 ppb, O_3_: 0–150 ppb) and once with higher fractions of NO_2_ (Figure S4b, CO: 0–400 ppb, NO_2_: 0–150 ppb, O_3_: 0–150 ppb). Recall that the generated fractions were sampled from uniform distributions. Due to the linearity of the model, the signal distributions are uniform as well, with two exceptions. The NO_2_-B4 sensor shows mainly noise for low fractions and is therefore close to normally distributed (Figure S4a), which becomes uniform for higher fractions once the signal-to-noise ratio increases (Figure S4b). The opposite is true for the OX-B4 sensor, but the reason is different. For low fractions of NO_2_, the sensor signal is mainly generated by O_3_. If higher amounts of NO_2_ are used, moderate sensor signals are generated in three different ways: low O_3_ and high NO_2_ values, high O_3_ and low NO_2_ values, or intermediate values of both gases. These three scenarios are more likely than the extremes responsible for the lowest and highest sensor signal intensities, i.e., very low and very high fractions of O_3_/NO_2_, leading to a triangular distribution.

**Figure 4:**
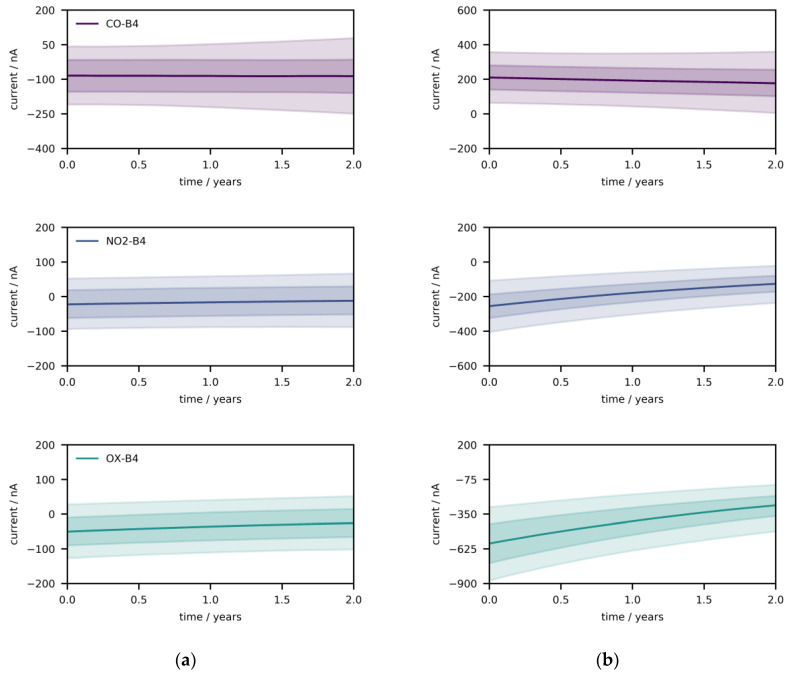
Distribution of sensor signals (q_0.50_, q_0.25–0.75_, q_0.05–0.95_) over time under constant conditions. (**a**) At low fractions (CO: 50 ppb, NO_2_: 50 ppb, O_3_: 50 ppb), the baseline drift is mildly recognizable (particularly for CO-B4). (**b**) At high fractions (CO: 600 ppb, NO_2_: 600 ppb, O_3_: 600 ppb), the curvature due to the sensitivity drift is clearly visible (particularly for NO_2_-B4 and OX-B4); this drift leads to convergence towards the baseline.

In a second step, 10,000 Monte Carlo experiments of sensor sampling, followed by monitoring over two years at moderate but constant fractions, were performed (Figure 4a, CO: 50 ppb, NO_2_: 50 ppb, O_3_: 50 ppb). Figure 4a depicts the population distribution (q_0.50,_ q_0.25–0.75_, q_0.05–0.95_) of the three sensor signals over time; the zero drift is mildly visible, especially for CO-B4. The same procedure is repeated at higher fractions (Figure 4b, CO: 600 ppb, NO_2_: 600 ppb, O_3_: 600 ppb). The signals have the tendency to converge towards the baseline (especially NO_2_-B4 and OX-B4), which can be attributed to the exponential decay of the sensitivity. In Figure S5a,b, one randomly sample device is examined, depicting noise, zero, and sensitivity drift. The trajectories show baseline drift at fractions of zero (Figure S5a), and the decay of sensitivity is pronounced at increased fractions of gases (Figure S5b). Overall, the zero drift appears to play a major role at low fractions, while the opposite is true for the sensitivity drift. The results are consistent with the data in Table 1.

**Figure 5:**
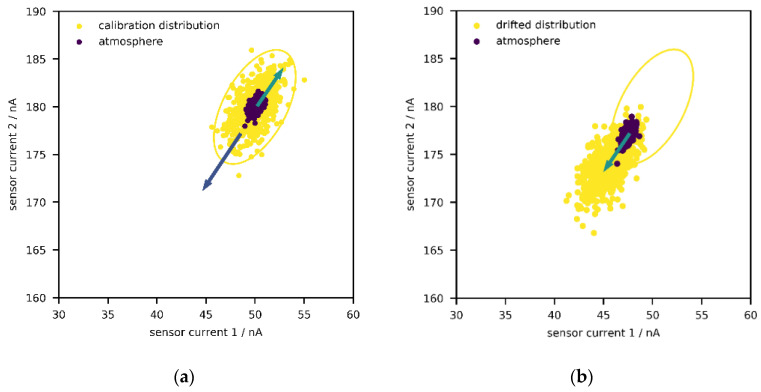
Distribution of sensor currents for a system consisting of two sensors. (**a**) Direction of aging drift (blue) is opposite to trajectory of atmosphere (green); trajectories are along largest expansion of the envelope. Signals decrease due to aging, but atmospheric amount fractions increase. Thus, the aging process is masked. (**b**) After some time, the generated signal intensities will be low enough to be outside the envelope and identified.

Before moving onto the results of the population model, it is worth mentioning that establishing PD for low-cost sensors will also require studying the dynamics of the atmosphere, i.e., trajectories and time constants of changes in fractions of the measured pollutants, and comparing it with the dynamics of aging over the whole population of sensors. As discussed above, field calibration captures only a (random) subset of the complete atmosphere at a specific location, and the relationships between the compounds change over time. Consequently, the current atmosphere can be interpreted as a smaller distribution within the field calibration distribution (Figure 5a). Depending on “how much” of the complete distribution has been captured during calibration, the current atmosphere might leave the envelope, which then causes anomalies, as seen in Figure 2.

Moreover, depending on the direction of the largest expansion of the envelope around the field data, the direction of drift in the signal space, and the trajectory of the atmosphere, the aging drift can be “masked” for some time. Such masking happens, for example, when the temporally local atmosphere, e.g., the collection of data points from the last week, moves towards higher levels (e.g., due to seasonality), thereby generating higher sensor signal intensities, while the aging drift leads to lower signal intensities, a scenario illustrated in Figure 5a. To elaborate this with a hypothetical example, suppose that the CO level was monotonically increasing over the course of a year, which would lead to a higher signal intensity; in addition, the sensor aging process was lowering the signal intensity over time. This would be interpreted as increasing gas levels at decreased speed (in comparison with reliable reference devices). Depending on the time constants of both processes, the signal distribution would shift a lot, although this would not be recognized, since the atmosphere would move in the opposite direction and would be still within the confidence region—i.e., inside the envelope. If aging drift carried on and the atmospheric gas levels finally decreased, the generated sensor signals would be low enough to be outside the envelope, thereby being detected by the ML algorithms at this point (Figure 5b).

Although such peculiarities delay the necessary maintenance, they are unlikely to happen on a regular basis. Another event is more probable and not limited to field calibration but can also occur with laboratory calibration. More precisely, if drift caused higher signal intensities over time, but the calibration limits are set much higher than what was observed in the atmosphere, the signal distribution could shift inside the envelope and no anomalies would be recognized over time, since the highest possible signal intensities would be always within the envelope.

Even if the upper calibration limits were not excessively high, the aging process can be hidden. Under the assumption that the population model reflects the real-word physics, the simulations above show that the existence of sensitivity drift leads to a compression of the distribution towards the baseline, i.e., the lowest possible value, while the baseline drift causes the baseline to move in any direction. Therefore, zero drift can be interpreted as a random vector with independent components acting on the baseline. As for sensitivity drift, another random vector acts on the maximum possible sensor signal and points towards the baseline, thus compressing the whole sensor signal distribution. 

**Figure 6:**
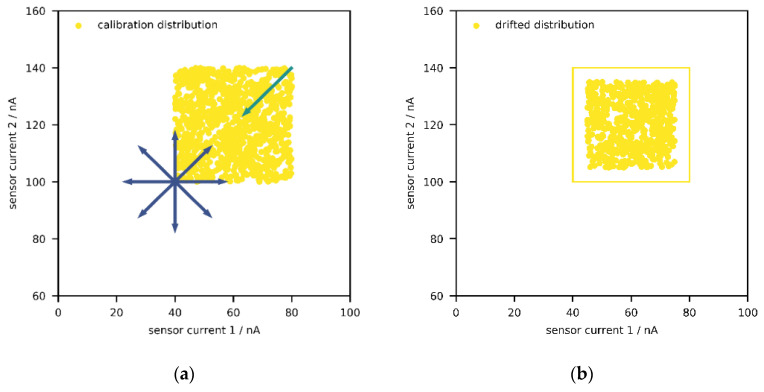
Distribution of sensor currents for a system consisting of two sensors. (**a**) Zero drift (blue) moves the baseline (lowest possible signal intensity) in any direction, while sensitivity drift (green) causes the maximum possible signal intensity to decay exponentially. (**b**) Example of a signal distribution that has shifted inside the envelope, thus no anomalies were detected.

With two sensors, the confidence region can be thought of as square-like region in two dimensions due to the uniform calibration distributions, and the baseline becomes the lowest possible value, given that fraction of zero is included in the calibration (Figure 6a). As mentioned above, issues might occur if the (shrinking) signal distribution shifts/drifts inside the envelope (Figure 6b), which might happen for a non-negligible portion of low-cost sensor systems. From geometrical interpretation, one could assume that the probability to drift inside the confidence region should be around 25% (one out of four squares). With three sensors, this probability then reduces to 12.5%, since there are eight cubes and the envelope is one of them, and the probability decrease further with the inclusion of further sensors. Hence, for a device with *p* sensors, this probability would be around 2^−*p*^. The outcome of such a process is that, over time, all incoming measurement results will be accepted and no anomalies detected anymore. Thus, there might be a subpopulation of devices failing to perform PD with the presented algorithms.

**Figure 7:**
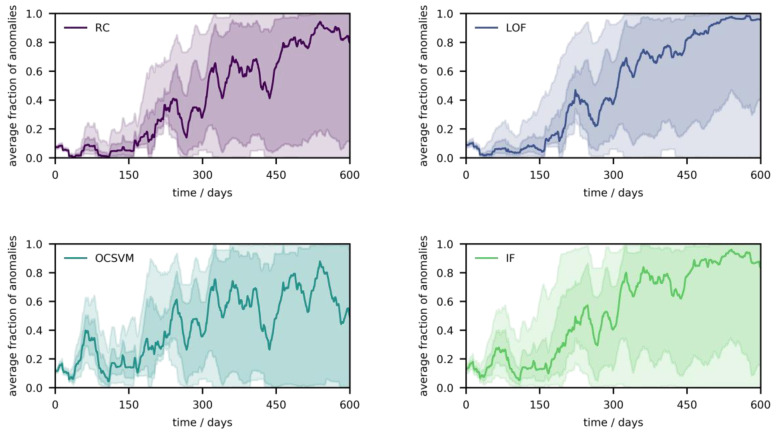
Performance of the four ML algorithms (*ν* = 0.10) as distribution of anomalies (monthly moving average) from the population of low-cost sensor systems (q_0.50_, q_0.25–0.75_, q_0.05–0.95_), computed in Monte Carlo simulations including laboratory calibration followed by aging.

Finally, to evaluate the performance of the four algorithms on the whole population of low-cost sensor systems with laboratory calibration, Monte Carlo experiments were combined with unsupervised anomaly detection (*ν* was fixed to 0.10 for consistency). In total, 1000 random devices were sampled and calibrated with 10,000 randomly sampled fractions (CO: 0–1000 ppb, NO_2_: 0–100 ppb, O_3_: 0–100 ppb), whereas the resulting signal distribution was used to train the algorithms. For drift monitoring, the data set of collected references values by Zimmerman et al. was iterated five times (i.e., repeating the four months of observations), thereby simulating pseudo-seasonality. These reference values were used to generate sensor signals and the devices underwent aging with increasing time index (according to Equations (4)–(6)), whereas the generated sensor signals were assessed according to their position relative to the envelopes. Figure 7 shows anomalies for the four algorithms (monthly moving average) over the whole population of low-cost sensor systems (q_0.50,_ q_0.25–0.75_, q_0.05–0.95_).

In the initial phase, the spread is small and becomes larger over time, as the influence of drift (i.e., its direction relative to the envelope) starts playing a role. The dynamics of the atmosphere are visible, as the fraction of anomalies increases and decreases periodically, although the median trend is to increase over time. In contrast to field calibration (Figure 2a and Figure S2), the recalibration frequency appears smaller when lab calibration is performed, which comes from the fact that the average fraction of anomalies peaks much later (Figure 2a and Figure 7), which can be explained by the extended calibration range. The differences between the four algorithms seems negligible; OCSVM and IF detect an anomaly peak in the half-time of the first 150 days, which is not the case for RC and LOF. However, this first anomaly peak has a much lower magnitude and occurs later in time compared with this hypothetical laboratory calibration, which is natural as no concept drift is possible. In this example, sensor aging becomes visible after five months as the four algorithms depict the increase in anomalies, though with a diverging anomaly fraction over the population.

While the PD approach with the presented algorithms appears to work for at least 50% of all devices (in 50% of all cases, the fraction of anomalies increases over time), it seems not to be true for at least some other 25% of the devices. This might support the aforementioned hypothesis since the trajectories of the lower quantiles indicate breakdown of the anomaly detectors. Remarkably, the probability of failing appears to be at least twice as high compared to what is calculated above (for a device with three sensors). On one hand, there is certainly a small portion of devices with little to no drift at all. On the other hand, even if the baseline does not move strictly inside the envelope but passes close to it, some part of the drifted signal distribution will overlap with the envelope, and depending on the dynamics of the atmospheric gas levels, signal drift can be masked again. The underlying issue is that drifts and changes in fractions are often indistinguishable without knowing any further information, thus it is of importance to engineer solutions that deal with this problem while still only having need of calibration data. Nonetheless, there is a chance that breakdown occurs less frequently in real-world scenarios, e.g., as some of the assumptions might not be fulfilled, thus field experiments would be needed to verify the results.

In addition to cost effectiveness, this also explains the preference towards preventive maintenance such as drift compensation using the combined knowledge of the whole sensor network. For example, a class of statistical methods have been developed that aim to detect and remove sensor drift components during operation (blind calibration), although this requires dense deployment of the network and an initial model learning phase [44,45,46]. Due to the reconstruction of the original signals, one initial laboratory calibration would be sufficient and no further recalibration would be needed. However, these methods are not necessarily dedicated to low-cost electrochemical sensors, as they assume random walk behavior of zero drift (which is more realistic) but tend to neglect sensitivity drift. Moreover, the approach becomes unstable if many sensors express drift, but also if the underlying pollution phenomena change over the year. 

If mobile sensor systems (e.g., on public transport or drones) with high measurement accuracy are deployed as well, frequently exchanged measurement results can also be used to perform cheap recalibrations without explicitly having to recognize drifts or the optimal moment for recalibration [47,48,49]. Similar approaches have been explored with stationary reference stations as recalibration seeds, although so far only for particulate matter [50]. Nonetheless, a handful of well-characterized low-cost sensor systems distributed across a city might do a similar job. On a final note, the question arises to what extent the different procedures could be superimposed to obtain even more reliable measurement results.

## 4. Conclusions and Outlook

This study examined the viability of four algorithms from unsupervised anomaly detection for PD of low-cost electrochemical sensor systems in air quality monitoring applications to optimize recalibration intervals and increase the reliability of measurement results, which will be particularly relevant for the operation of large sensor networks. Such approaches are particularly interesting as they rely purely on calibration data, which is available right away after characterization.

The performed experiments have shown that devices that are calibrated on the field can yield untrustworthy measurement results over time and might require shorter recalibration times due to concept drift, which superimposes with drift due to sensor aging. Nonetheless, anomaly scores can disclose the reliability of the measurement results. On one hand, by combining laboratory calibration with drift compensation methods, concept drift could be removed from the software side. On the other hand, reduction in unit-to-unit variability and sensor drifts should be prioritized from the hardware side since it would enable population calibration without any further recalibration.

Moreover, the four discussed methods from unsupervised anomaly detection recognize drifts most of the time, but simulation results suggest that they might fail for a moderate portion of low-cost sensor systems, as sensor variability and joint dynamics of the drift components and atmosphere complicate the approach. In the selection of a suitable solution, interactions between all potential drift components and unit-to-unit variability have to be considered. Therefore, the obtained results can guide the development of novel algorithms and the establishment of methodologies that are adapted to the physics of low-cost electrochemical sensors and the calibration approach. In particular, network calibration techniques for electrochemical sensor systems could be explored in future studies on both real-world data and simulations such as the ones proposed.

## Figures and Tables

**Table 1:** Properties of the electrochemical gas sensors.

	Sensitivity nA/ppm	Zero nA	Sensitivity Drift (1-%Change/100)/Year	Zero Drift nA/Year
CO-B4	0.42–0.65	−250.00–30.00	0.90–1.00	−50.00–50.00
NO_2_-B4(3F)	−0.65–−0.20	−80.00–80.00	0.60–0.80	−12.00–12.00
OX-B4(31) ^1^	−0.75–−0.23	−80.00–80.00	0.60–0.80	−12.00–12.00

^1^ Treats NO_2_ and O_3_ equally.

**Table 2:** Summary statistics (quantiles) of calibration distribution inside envelope and monitoring distribution outside envelope.

		q_0.01_	q_0.05_	q_0.25_	q_0.50_	q_0.75_	q_0.95_	q_0.99_
**CO/ppb**	c	99.0	107.5	148.8	182.0	243.1	416.7	558.5
	m	138.8	216.0	299.0	515.7	819.1	1472.0	1861.7
**NO_2_/ppb**	c	1.0	2.4	4.7	7.2	9.9	16.7	20.5
	m	10.1	11.4	11.4	23.7	28.3	35.3	43.0
**O_3_/ppb**	c	5.4	9.3	16.6	23.1	30.5	42.1	47.8
	m	0.1	0.8	5.5	21.1	21.1	38.0	51.9

## Data Availability

The developed code can be found on GitHub (metas-ch/AlphaDrift).

## Supplementary Materials

The following are available online at https://www.mdpi.com/article/10.3390/s21093298/s1: Figure S1: Structure of the used data set, values scaled to the range between zero and one. White regions refer to missing data, whereas most missing values are present in sensor data of devices 2 and 3. Figure S2: (a) Fraction of anomalies (weekly moving average) over four months for different ν across algorithms after two weeks of field calibration for sensor data of device 2. (b) Fraction of anomalies (weekly moving average) over four months for different *ν* across algorithms after two weeks of field calibration for sensor data of device 3. Figure S3: (a) Fraction of anomalies (monthly moving average) over a year for different *ν* across algorithms after two weeks of field calibration for sensor data. (b) Fraction of anomalies (monthly moving average) over a year for different ν across algorithms after two weeks of field calibration for reference data. Figure S4: Calibration results for one randomly sampled device (exposure to 10,000 uniformly sampled gas concentrations). Calibration distribution, i.e., distribution of the target gases, is uniform and so are sensor signals, though with two exceptions. For low fractions of NO_2_ (CO: 0–400 ppb, NO_2_: 0–15 ppb, O_3_: 0–150 ppb), the NO_2_-B4 signal is composed of normally distributed noise. For high fractions of NO_2_ (CO: 0–400 ppb, NO_2_: 0–150 ppb, O_3_: 0–150 ppb), the signal of the OX-B4 sensor becomes triangularly distributed. Figure S5: (a) Trajectories of sensor signals at lower concentrations of target gases (CO: 50 ppb, NO_2_: 50 ppb, O_3_: 50 ppb) for a randomly sampled device; drift of baseline is clearly visible. (b) Trajectory of sensor signals at higher concentrations of target gases (CO: 600 ppb, NO_2_: 600 ppb, O_3_: 600 ppb) for a randomly sampled device; drift of sensitivity is visible and causes some curvature of the trajectory.
