# Supplementary material for: Relevance of Drift Components and Unit-to-Unit Variability in the Predictive Maintenance of Low-Cost Electrochemical Sensor Systems in Air Quality Monitoring
Source: Sensors (Basel). 2021 May 10;21(9):3298. doi: 10.3390/s21093298 (PMC8126229; doi:10.3390/s21093298)
Supplement: Supplementary file 1 [file sensors-21-03298-s001.zip › sensors-1160014-supplementary.pdf]

Supplementary Materials

Relevance of Drift Components and Unit-to-Unit Variability in the Predictive Maintenance of Low-Cost Electrochemical Sensor Systems in Air Quality Monitoring

Georgi Tancev

Swiss Federal Institute of Metrology, 3084 Bern, Switzerland; georgi.tancev@metas.ch

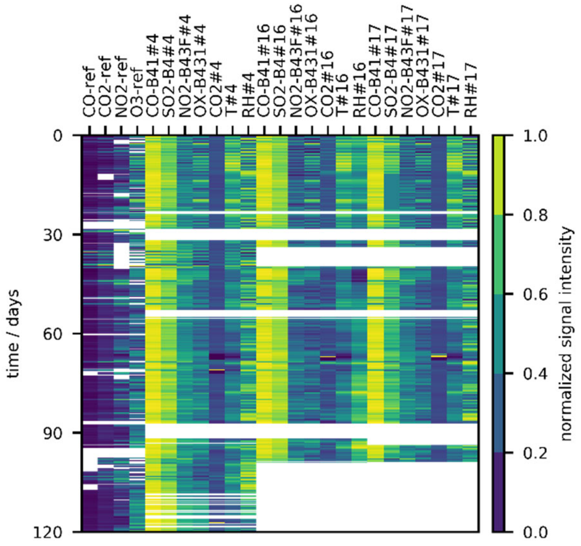

Figure S1. Structure of the used data set, values scaled to the range between zero and one. White regions refer to missing data, whereas most missing values are present in sensor data of devices 2 and 3.

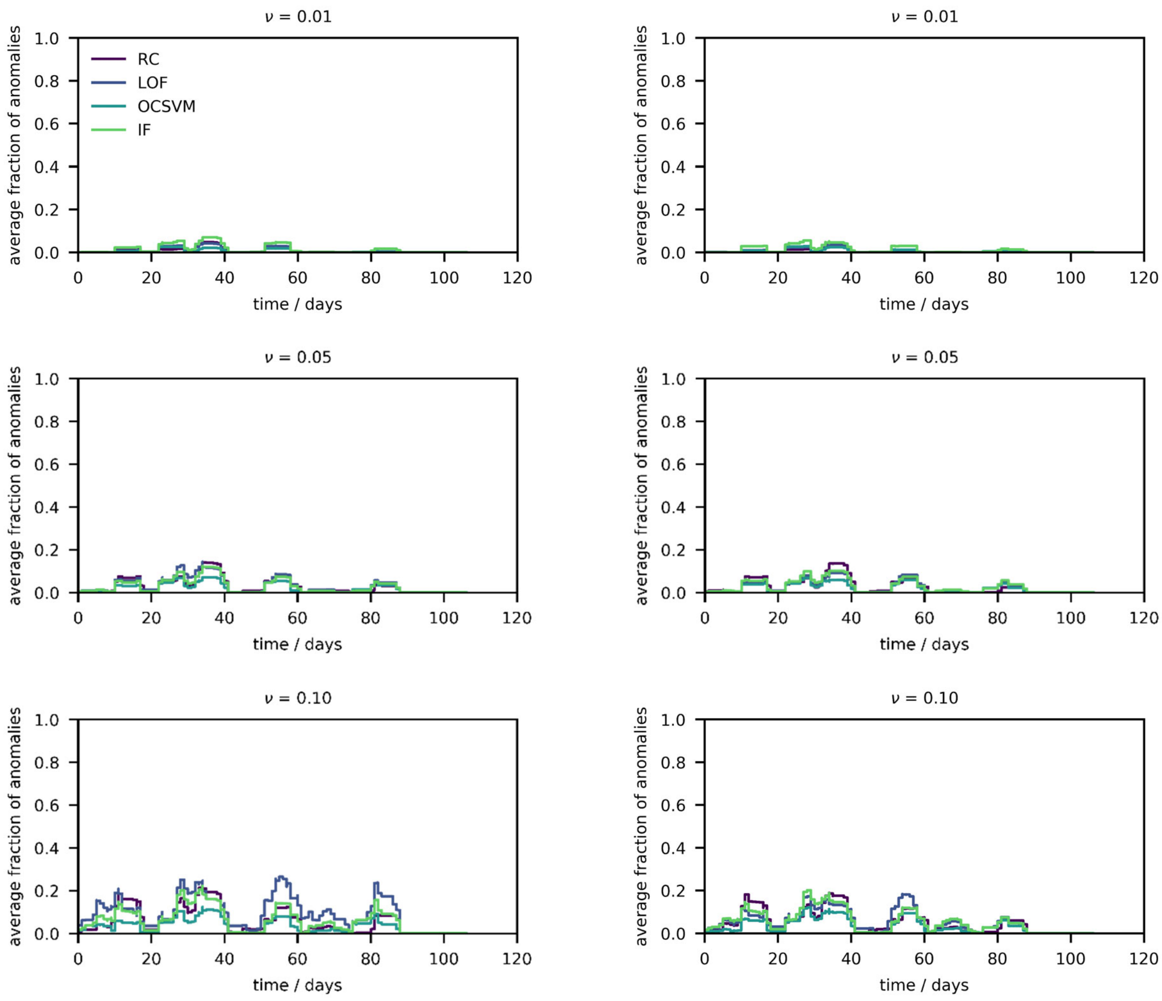

(a) **(b)**

Figure S2: **(a)** Fraction of anomalies (weekly moving average) over four months for different ν across algorithms after two weeks of field calibration for sensor data of device 2. **(b)** Fraction of anomalies (weekly moving average) over four months for different ν across algorithms after two weeks of field calibration for sensor data of device 3.

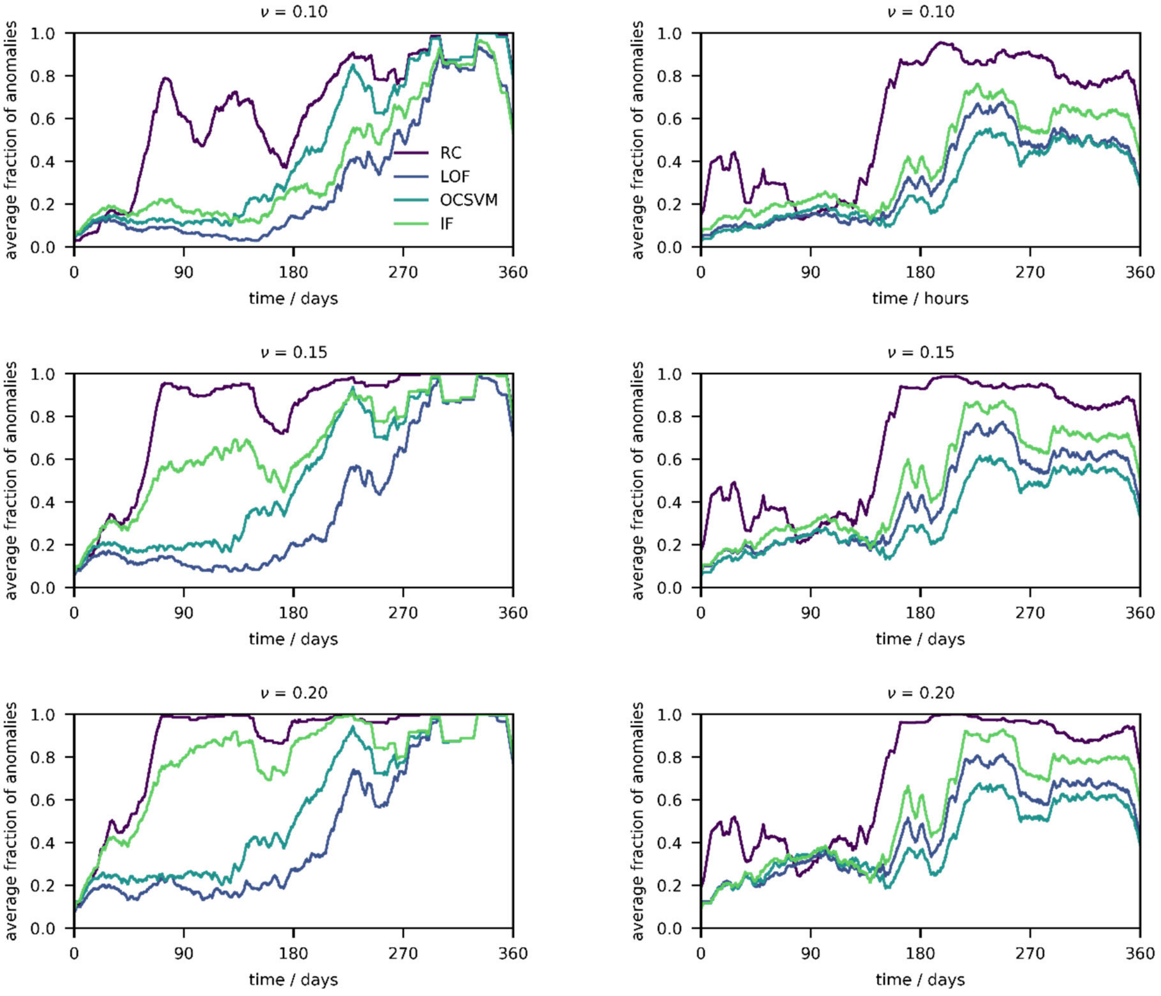

(a) **(b)**

Figure S3: **(a)** Fraction of anomalies (monthly moving average) over a year for different ν across algorithms after two weeks of field calibration for sensor data. **(b)** Fraction of anomalies (monthly moving average) over a year for different ν across algorithms after two weeks of field calibration for reference data.

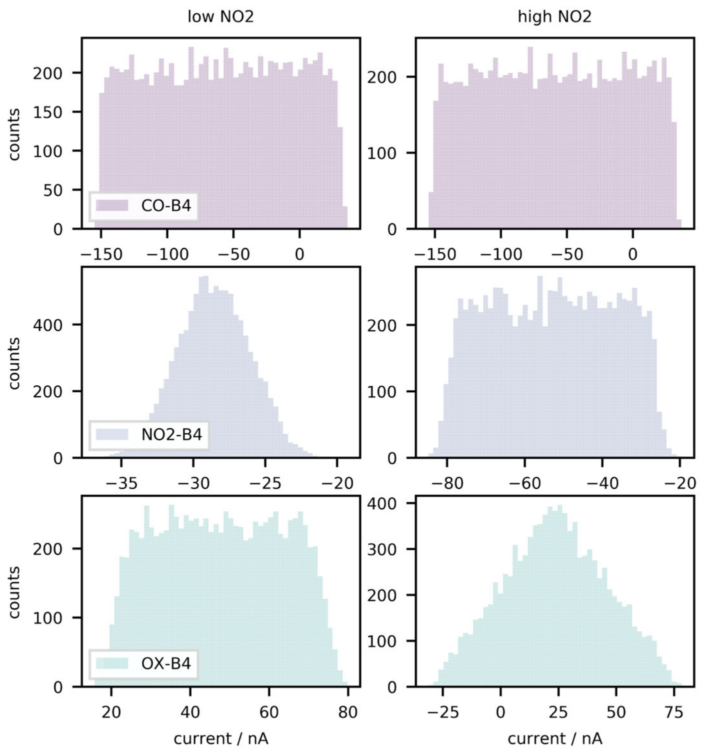

Figure S4: Calibration results for one randomly sampled device (exposure to 10,000 uniformly sampled gas concentrations). Calibration distribution, i.e., distribution of the target gases, is uniform and so are sensor signals, though with two exceptions. For low amount fractions of NO_2 (CO: 0-400 ppb, NO_2: 0-15 ppb, O_3: 0-150 ppb), the NO_2-B4 signal is composed of normally distributed noise. For a high amount fractions of NO_2 (CO: 0-400 ppb, NO_2: 0-150 ppb, O_3: 0-150 ppb), the signal of the OX-B4 sensor becomes triangularly distributed.

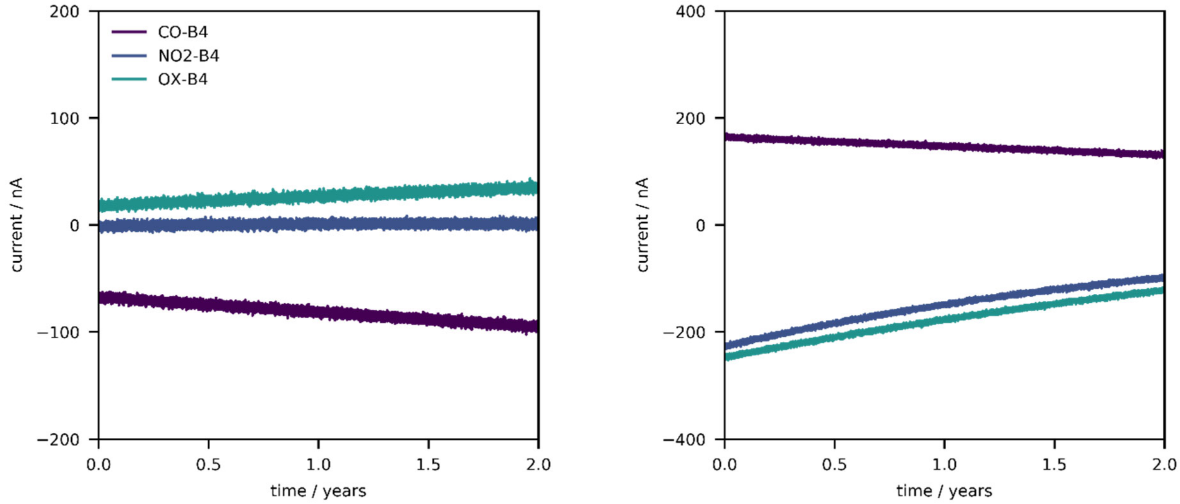

(a) **(b)**

Figure S5: (a) Trajectories of sensor signals at lower concentrations of target gases (CO: 50 ppb, NO_2: 50 ppb, O_3: 50 ppb) for a randomly sampled device; drift of baseline is clearly visible. **(b)** Trajectory of sensor signals at higher concentrations of target gases (CO: 600 ppb, NO_2: 600 ppb, O_3: 600 ppb) for a randomly sampled device; drift of sensitivity is visible and causes some curvature of the trajectory.
